# Continuous video recording with simultaneous amplitude-integrated EEG monitoring to improve seizure recognition in newborns

**DOI:** 10.3389/fped.2025.1585606

**Published:** 2025-06-04

**Authors:** Anup Kage, Yesenia C. Santana, Ivone L. Malcolm, Caroline Francia, Michael Yoong, David Wertheim, Divyen Shah

**Affiliations:** ^1^ Blizzard Institute, Centre for Genomics, Queen Mary University of London, London, United Kingdom; ^2^ Neonatal Intensive Care Unit, The Royal London Hospital, London, United Kingdom; ^3^ School of Computer Science and Mathematics, Kingston University, Kingston upon Thames, United Kingdom

**Keywords:** newborn seizures, aEEG, EEG, video recordings, abnormal movements

## Abstract

**Introduction:**

Seizures in newborn infants are considered a neurological emergency requiring prompt treatment to limit exacerbation of brain injury. Digital monitors incorporating limited channel raw EEG and amplitude-integrated EEG (aEEG/EEG) are widely used. This study aimed to determine if continuous video recording with simultaneous aEEG/EEG recording enhances seizure recognition.

**Methods:**

Newborns at risk of seizures who underwent neuromonitoring with aEEG/EEG were prospectively recruited to an observational study in a tertiary neonatal centre. Video recordings were commenced after obtaining written consent from parents.

**Results:**

Simultaneous video recordings with aEEG/EEG were obtained in 15/47 newborns recruited to study. A total of 116 electrographic seizure episodes were detected on aEEG/EEG when a total of 56 episodes of abnormal movements were noted on video recordings. Only 8 of these abnormal movements had simultaneous electrographic seizures on aEEG.

**Conclusion:**

Use of simultaneous video and aEEG/EEG recordings in newborns at risk of seizures is feasible. It not only assists confirmation of the presence of seizures but may also help in identifying movements associated with abnormal neurology that are not seizures.

## Highlights

• Continuous video recording with simultaneous aEEG/EEG monitoring is straightforward, feasible and aids interpretation of abnormal movements.
• Video recording has the potential to improve accuracy of seizure recognition and aid differentiating seizures from non-seizure abnormal movements.

## Background

The neonatal period is the most vulnerable for developing seizures, particularly in the first week after birth (1). Seizures in the newborn are considered a neurological emergency and they are often associated with underlying pathology of the developing brain (2). Prompt recognition and evaluation is necessary for timely treatment in order to terminate the seizure activity and limit or prevent brain injury (3). High seizure burden is known to be associated with cerebral tissue injury on MR imaging (4) and increases the likelihood of an adverse outcome.

Abnormal neurologic movements may occur in babies with release of the brain stem from inhibitory cortical influence (5) in conditions such as hypoxic-ischemic encephalopathy (HIE) and these may be mistaken for clinical seizures as may tremors and jitteriness. Most clinical seizures in newborns are subtle (6) and as such are easily missed (7). Continuous multichannel-video electroencephalography (EEG) is considered to be the gold standard for monitoring and detection of neonatal seizures (8). However, it is resource intensive, and availability is limited. Monitors with two channels of amplitude-integrated EEG (aEEG) and raw EEG (from here on annotated as aEEG/EEG) are widely used on neonatal units for continuous monitoring (9) whereas in the UK over half of the units having no access to multichannel EEG out of hours (10).

Optimal seizure treatment in the newborn presents multiple challenges. The use of established anticonvulsants in newborns is based on evidence from relatively few randomised controlled trials (11). The subtle nature of clinical seizures in the newborn puts the patients at risk of over and undertreatment. Seizures, particularly when first suspected, are commonly treated prior to commencing aEEG/EEG monitoring. As a result, newborns with abnormal movements that may not be seizures may receive antiseizure medication.

Variane et al. (12) have demonstrated the feasibility of video with aEEG as a model of “telehealth” and remote neuromonitoring in a low- or middle-income setting in newborns with HIE receiving therapeutic hypothermia. Although various commercial systems incorporating video with multiple channels of EEG are available, the simultaneous use of continuous video in combination with a dedicated aEEG/EEG monitor is not routinely used in UK neonatal centres for monitoring of seizures in newborns. We hypothesized that the use of video can aid distinction of abnormal movements associated with EEG seizures from those that are not related to seizures.

Thus, the aims of this study were to determine the feasibility of simultaneous continuous video recordings with aEEG/EEG monitoring and whether the combination can improve recognition of seizures in newborns.

## Methods

Newborns at risk of seizures or suspected of having seizures were enrolled into the ADAPTS (Automated Detection and Prediction of Term newborn Seizures) Study after signed informed consent was obtained from parents. This was a prospective single centre observational study carried out at the Royal London Hospital, Barts Health NHS Trust, London between June 2021 and August 2023. aEEG/EEG recording was commenced on newborns as per local clinical practice.

Seizures were treated by attending clinicians based on clinical presentation with additional information from the aEEG/EEG as per local clinical guidelines; phenobarbitone (up to 40 mg/kg) was used as the first line anti-seizure medication followed by phenytoin (18 mg/kg) or midazolam (bolus of 150 µg/kg followed by 30–150 µg/kg/hr continuous infusion). Levetiracetam at a loading dose of 18 mg/kg followed by maintenance dose of 5 mg/kg BD is used as next line of anti-seizure medication.

Ethics approval (research ethics committee reference 20/PR/0969) was granted for retrospective use of the aEEG/EEG recordings subject to parental consent. Ethics approval was granted for prospectively commencing video recording and use for the study only after written consent was obtained from parents.

aEEG/EEG recordings were carried out using the Olympic Brainz Monitor v3.1.5 (Natus Medical Incorporated, Middleton, USA), a 2-channel electroencephalography and cerebral function monitor system. With a reference electrode placed on left shoulder, the signals EEG were acquired from P3-P4, C3-P3, enabling display of C4-P4 channels also.

A Logitech C920s camera with Kaltura software (Kaltura Inc., New York, USA) was used to capture video recordings onto a laptop (Dell Technologies, USA). The videos were acquired with the resolution set at 1080p HD and frame rate of 10 frames per second (fps). The camera was mounted on a tripod and placed at the foot end of the cot. The clocks on the laptop and aEEG/EEG monitors were synchronized at the start of the study.

Amplitude integrated EEG (aEEG) recordings together with raw EEG were assessed offline independently by two experienced neonatal consultants (DKS and ACK) both of whom were blinded to clinical details. Only unequivocal seizures were included in the analysis. A seizure was defined as evolving rhythmic spike and wave activity lasting for at least 10 s on the raw EEG in the absence of artefact (13,14). As in our previous study (4), seizure burden was classified as high if seizures occurred for more than 15 min in any one hour period (frequent seizures) or more than 30 min in any 1 h period (status epilepticus) within a 24 h epoch. Low seizure burden was defined as seizures lasting for less than 15 min within an hour (sporadic seizures), or no seizures.

Video recordings were assessed by YCS off-line for abnormal movements and seizures without knowledge of clinical information about the infants. The assessments of individual abnormal movement episodes were confirmed by DS and ACK.

## Results

### Patient recruitment

**Figure 1:**
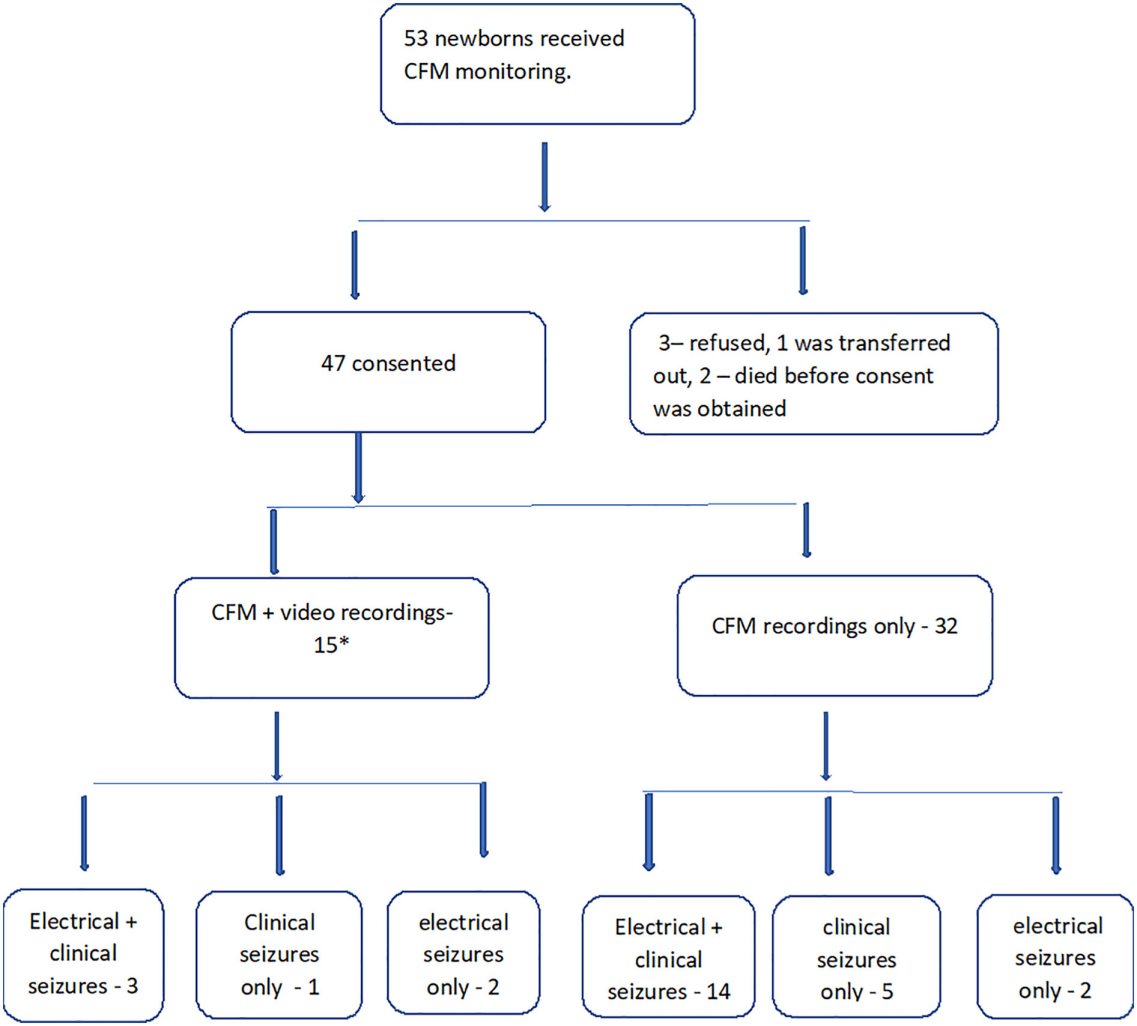
Flowchart of the study population. *In 3 newborns, seizures ceased before video recording could be commenced.

Of 47 babies recruited into the study, consent was obtained for video recordings from 15 (Figure 1). Twenty-five families opted out of video recording, one died, aEEG/EEG monitoring ended in five before video could be commenced and one was transferred to another hospital before consent could be obtained.

### Video recordings

The demographics of the 15 term newborns who had video recordings are shown in Table 1. Nine of 15 (60%) were suspected to have clinical seizures by treating clinicians. Eight of these nine had electrographic seizures noted on the aEEG/EEG. In three of these eight, electrographic seizures ceased before video recordings could be commenced. Thus, there were five infants who had simultaneous aEEG and video recordings during episodes of EEG seizures.

**Table 1:** Perinatal characteristics of the 15 newborns with electrographic seizures expressed as median (interquartile range).

Characteristics	Median (IQR)
Gender: Male *n* (%)	11 (73%)
Female *n* (%)	4 (27%)
Birth weight in grams	3,305 (2,885–3,455)
Gestational weeks	38 (37–39)
Apgar score at 1 min	1 (1–3)
5 min	4 (2–7)
10 min	6 (4–8)
First pH	7.13 (6.91–7.29)
Lactate	9 (5–14)
Newborns with seizures identified on aEEG/EEG *n*(%)	8 (53%)
Clinical seizures *n* (%)	9 (60%)
Anticonvulsant medications *n* (%)	11 (73%)
Ventilated *n* (%)	10 (67%)
HIE diagnosis *n* (%)	8 (53%)
Therapeutic hypothermia *n* (%)	8 (53%)
Survived to discharge home *n* (%)	14 (93%)
Normal neurological examination at discharge *n* (%)	8 (53%)

A total of 1,100 h of video were reviewed. The median (IQR) age at commencing video recording was 48 (25 to 66) hours. The median (IQR) duration of video recordings was 76 (59 to 97) hours and the median (IQR) interval between commencing aEEG/EEG monitoring and video was started, and 19 (8 to 43) hours respectively.

Five newborns had electrographic seizures while video recording was ongoing; three newborns had electrographic seizures with clinical manifestations on the video recordings and two had only electrographic seizures. The charcteristics of these five newborns are noted on Table 2.

**Table 2:** Clinical characteristics of newborns with both electrical seizures and clinical seizures seen on video recordings.

Characteristics	Electrical and clinical seizures on video and aEEG			Electrical seizures seen on aEEG	
	Newborn 1	Newborn 2	Newborn 3	Newborn 4	Newborn 5
Seizure burden	High	Low	High	High	High
Electrocortical background	Continuous normal voltage	Normal	Discontinuous	Burst suppression	Burst suppression
Clinical manifestations	Subtle seizures (mouth and tongue movements) that progressed to clonic seizures involving the upper extremities	Subtle seizures including eye fixation and mouth gestures followed by clonic movements of the upper and lower extremities	Myoclonic seizures	No clinical symptoms	No clinical symptoms
Anticonvulsant drugs used	Phenobarbitone, midazolam, phenytoin	Phenobarbitone	Phenobarbitone and midazolam	Phenobarbitone and midazolam	Phenobarbitone, phenytoin and midazolam
Diagnosis	Bilateral stroke	HIE	HIE	HIE	HIE
Therapeutic hypothermia	No	Yes	Yes	Yes	Yes

During simultaneous aEEG and video recording, a total of 116 seizure episodes were diagnosed on aEEG when a total of 56 episodes of abnormal movements were picked up on video recordings. Only 8 of these abnormal movements had simultaneous electrographic seizures on aEEG.

Three newborns were identified to have abnormal movements on video recordings with no corresponding electrographic seizure activity on aEEG/EEG (Table 3).

**Table 3:** Clinical characteristics of newborns with no electrical seizures but abnormal movements noted on video recordings for which anti-seizure medications were administered.

Characteristics	Newborn 1	Newborn 2	Newborn 3
Clinical manifestations	Mouthing and repetitive tongue movements with cycling movements of arms and legs	Myoclonus	Myoclonus
Use of anticonvulsant drugs	Yes, phenobarbitone	Yes, phenobarbitone	No
Diagnosis	Hyponatremia	HIE	Anorectal malformation
Therapeutic hypothermia	No	No	No

## Videos in Supplementary Material

With written parental permission, excerpts of video recordings with the corresponding aEEG/EEG are included online (Supplementary Videos S1, S2).

Electronic Supplementary Video S1: Baby was born at term gestation by normal vaginal delivery with birth weight of 3,480 g. He was on the postnatal ward with mother when at 18 h of age, was noticed to have left sided jerky movements of upper and lower limbs with lip smacking. He was admitted to the neonatal intensive care soon after. At 19 h of age, baby was having slow rhythmic movements easily seen in proximal muscles of the left upper limb with associated twitching movements in the eyes and oro-buccal movements. Towards the end of the video, rhythmic movements are also seen in the right upper limb. The corresponding aEEG trace shows a rise in lower and upper margin. The corresponding raw EEG shows rhythmic high voltage spike and wave activity. He was loaded with phenobarbitone at 20 mg/kg.

Electronic Supplementary Video S2 shows a baby born at term gestation by normal delivery weighing 3,430 g. He was admitted to the neonatal intensive care unit from the postnatal ward at 9 h of age following dusky episodes. In the video, baby is having oro-buccal movements with alternating cyclical movements of the upper and lower limbs. The corresponding aEEG shows continuous normal voltage trace. The raw EEG shows no features of seizures.

## Discussion

Our study demonstrates that simultaneous use of video with the aEEG/EEG monitoring in newborns at risk of seizures is feasible. Although video is routinely available on multichannel raw EEG systems, it’s availability on routinely used aEEG/EEG monitors is more limited and to our knowledge video with aEEG monitoring outside of research settings in not used in the UK.

Once consent was obtained, we were able to carry out video recording easily, with continuous unobtrusive recording at the bedside causing no apparent inconvenience in providing the regular ongoing medical and nursing care. The resolution and sampling rate used for recording the video were sufficient to enable recognition of seizure activity and, in combination with the aEEG/EEG, allowing distinguishing abnormal movements from those not considered to be seizures by an expert. The approach allowed confirmation of seizure activity in three newborns and identification of abnormal non-seizure movements in three newborns.

Although multiple channel EEG monitoring equipment may include an inbuilt camera, in our study we have used standalone aEEG/EEG monitors with 2 channel aEEG recordings with which we combined with an external HD webcam connected to a laptop. Digital aEEG/EEG monitors comprising limited channel aEEG with EEG are commonly used to monitor continuous brain activity using one or two channels (15). An international web based survey predominantly covering Europe and the USA reported that 65% of neonatal unit respondents used aEEG monitoring, exclusively in 20% of units and as an adjunct to EEG monitoring in a further 45% (16). Effectiveness of limited channel aEEG/EEG for seizure detection has been studied with sensitivities for individual seizures in the range of 27%–84% and specificities of 92%–96% (17). The use of video concomitantly with the aEEG/EEG may not only improve seizure recognition but may also prevent over-diagnosis of seizures based on clinical manifestations alone, thereby decreasing the inappropriate exposure to anticonvulsant medications. Over treatment not only exposes newborns to the side effects of antiseizure medications but also prolongs hospital stay with the potential for undue parental separation and anxiety (18).

Phenobarbitone and phenytoin were introduced to treat seizures in adults and later extrapolated for use in newborns and children (19). In experimental models, phenobarbitone has been reported to have adverse effects on the developing brain, including inhibition of brain growth, neuronal toxicity and adverse behavioural and cognitive effects into adult life when administered to young animals (20). Phenytoin exposure in immature rat pups produced widespread neuronal death as a result of apoptosis (21). Phenytoin toxicity has also been reported to cause hypotension and cardiac arrhythmias in the clinic (22).

Undertreatment may lead to intensification of the seizure activity, further worsening pre-existing brain injury and altering seizure thresholds (18). Under treatment of seizures is associated with periods of hypoxia, cerebral oedema and variations in cerebral perfusion pressure (23). High seizure burden is also known to cause adverse neurodevelopmental outcomes including cerebral palsy, epilepsy and psychomotor deficits (24). Hence accurate cotside seizure detection is imperative not only to provide appropriate treatment, but also to optimise outcomes in newborns.

Simultaneous video recording with aEEG/EEG may also assist with the identification of abnormal movements related to abnormal neurology that are not seizures. Some of the motor phenomena that may mimic seizures in the neonatal period include tremors, jitteriness, myoclonus and rarely hyperekplexia (5). Tremors are involuntary, rhythmic, oscillatory movements with equal amplitude around a fixed axis and can be fine or coarse depending on the amplitude (25). Tremors and jitteriness are usually benign but may be pathological secondary to hypoglycaemia, hypocalcaemia, hypothermia and drug withdrawal (26). Many abnormal movements are thought to be caused by continuous muscle stretch reflex due to immature spinal inhibitory neurons and usually resolve with age and neuronal maturity (27). They can be differentiated from seizures as they can be elicited by external stimuli, stop when the affected limb is restrained and flexed and are not associated with epileptic discharges on EEG.

Myoclonus is a jerk like movement of the limb, irregular and arrhythmic caused by muscle contraction. It can be repetitive and bilateral, be benign or pathological. Benign sleep myoclonus is jerk like movements seen only during sleep and disappear when the newborn wakes up. Non-epileptic pathological myoclonus is seen in encephalopathies and intraventricular haemorrhage. This is usually seen in diffuse cerebral injury and is thought to arise due to cortical inhibition of normally suppressed brain activity (28).

In a focus group study (29) aimed at exploring perceptions of the use of live video recording in neonatal units amongst parents and health professionals, it was noted that common themes for both parental and professional groups were best interests of the child and improved outcomes. Parents believed if live video improved the care of the child, then it was acceptable. Data protection and privacy was also raised by parents with a constant fear of data being compromised when Wi-Fi was in use. A questionnaire-based study (30) reported that parents had positive attitudes towards implementation of a webcam system in the neonatal unit. Parents were less concerned about privacy risks and more confident of the security system when compared to health professionals.

Parents perceptions towards research studies involving video recordings may be different compared to use of video recordings as part of standard clinical care; this difference needs further exploration. In our study consent for the use of video recordings was obtained in 15 out of 47 newborns (32%) with more than two third of the families (32/47, 68%) opting out of video recordings. Video recordings in our study were carried out for research and were not as part of an established standard of clinical care.

## Limitations

The study was conducted at a single tertiary centre. The numbers are small due to the difficulties in obtaining consent for video recording as mentioned earlier. The video recordings were started at a median age of 48 h. This may have missed some of the clinical seizures which commonly occur within the first 48 h in neonates. It is also known that neonatal seizures are of short duration, low amplitude and may occur outside the regions that is covered by the aEEG/EEG electrodes. Hence, aEEG/EEG monitoring may not detect all electrographic seizures.

## Conclusions

We have demonstrated that the use of simultaneous continuous video with aEEG/EEG is straightforward, feasible and can help interpretation of abnormal movements in neonates. It is relatively easy to set up with little technical expertise needed and the equipment was not seen as obtrusive by nurses when attending to the clinical care of babies. This approach also has the potential to aid seizure recognition and identify those abnormal movements which may not be seizures. The addition of digital video recording can also aid remote and retrospective expert review and hence improve seizure recognition. Improved seizure recognition and treatment has the potential to improve long term outcomes with reduced brain injury and improved neurodevelopmental outcomes and alleviate healthcare costs. Large multicentre study is needed to demonstrate the effective use of video recording with aEEG/EEG monitoring.

## Data Availability

The original contributions presented in the study are included in the article/Supplementary Material, further inquiries can be directed to the corresponding author.
